# Wound Healing Activity of *Rubus sanctus* Schreber (Rosaceae): Preclinical Study in Animal Models

**DOI:** 10.1093/ecam/nep137

**Published:** 2011-06-23

**Authors:** Ipek Süntar, Ufuk Koca, Hikmet Keleş, Esra Küpeli Akkol

**Affiliations:** ^1^Department of Pharmacognosy, Faculty of Pharmacy, Gazi University, Etiler 06330 Ankara, Turkey; ^2^Department of Pathology, Faculty of Veterinary Medicine, Afyon Kocatepe University, 03200, Afyonkarahisar, Turkey

## Abstract

Young shoots of *Rubus* species have been used for healing of wounds, infected insect bites and pimples in folk medicine for ages. In order to evaluate the wound healing activity of *Rubus sanctus*, four different extracts were prepared from the whole aerial parts of the plant by using *n*-hexane, chloroform, ethyl acetate and methanol, respectively. Incision wound healing model by using tensiometer on rats and excision model on mice were employed to assess the activity. Remarkable wound healing activity was observed with the ointment formulation of the methanol extract at 1% concentration on the mentioned models. The results of histopathological examination also supported the outcome of both incision and excision wound models. The wound healing effect was comparatively evaluated with a reference ointment Madecassol. The experimental data confirmed the ethnobotanical usage of *R. sanctus*.

## 1. Introduction

*Rubus* species (*Rubus* sp.) (Rosaceae) have been traditionally used for therapeutic purposes. For instance, extracts of leaves and roots of this genus have been used for the treatment of diabetes mellitus, rheumatism, sore throat, hemorrhoid, diarrhea and similar enteric disorders [1–5]. Particularly, decoction prepared from the roots of *Rubus sanctus* Schreber is used as herbal tea to alleviate pain and to heal rheumatism [6]. Moreover, dried and crushed young shoots of *Rubus* sp. have been applied onto wounds, infected insect bites and pimples [7].

Several biological activity tests have been carried out so far under laboratory conditions on *Rubus* species, which focus on antimicrobial [8, 9], radical scavenging [10], anticonvulsant muscle relaxant [11] and antinflammatory and antinociceptive activities [12, 13]. According to phytochemical analysis the plant extract characterized by their capability of synthesizing and accumulating ellagitannins containing a sanguisorboyl group [14]. They have also been found to metabolise several phenolic carboxylic acids, such as ellagic acid, and phenyl propanoids, particularly caffeic acid [15]. In a previous study, the aerial parts of *R. sanctus*, *R. hirtus* Walds. et Kit and their hybrid were evaluated for their anti-inflammatory activity using carrageenan-induced hind paw edema on mice and polar fractions, *n*-butanol and remaining aqueous fractions obtained by solvent extraction, were shown to possess significant activity [12]. In a different study on *Rubus* sp., ethanolic extracts of *R. sanctus* (root and aerial part) and *R. hirtus* (aerial parts) showed potent antinociceptive activity, while that of aqueous extracts had weak [13]. However, it should be noted that in the same study both plants' extracts had tendency to induce gastric damage. Ongoing studies revealed the novel anti-inflammatory triterpenoids, tormentic acid and euscaphic acid, which were isolated from the fresh leaves of *R. sieboldii* Blume [16].

Furthermore, phytochemical studies exposed chemical content of the aerial parts of some *Rubus* sp., which contain flavonoids (quercetin, kaempferol, caffeic acid and chlorogenic acid), phenolic acids, tannins, amino acids, sugars, pectins, carboxylic acids, anthocyanins, catechins, vitamin C and saturated or unsaturated fatty acids [17–20].

A survey of the published literatures revealed that wound healing property of *R. sanctus* has not been subjected to *in vivo* investigation by using incision and excision models.

The goals of the pharmacology of wound healing are to evaluate the influence of various measures in wound management programs on healing and to screen drugs that encourage healing. Several candidates have so far been used and were declared to affect healing in various ways. Nevertheless, thorough research in wound healing has not yielded, economic and efficient pro-healing agent that could preclude the long hospitalization of patients following surgery and wound imposition. The present investigations were planned to study the wound healing activity of *R. sanctus* Shreber. We undertook the present activity screening study in order to evaluate traditional use of this plant in terms of scientific point. The *n*-hexane, chloroform, ethyl acetate and methanol extracts prepared from the aerial parts of *R. sanctus* were tested in rats and mice for wound healing activity via incision by using tensiometer and excision wound models.

## 2. Methods

### 2.1. Plant Material

*Rubus sanctus* Schreber aerial parts were collected from Kıbrısköy village, Ankara, Turkey during June to July, 2007. The plant was authenticated by Serdar Arslan from Gazi University, Department of Biology, Faculty of Science and Art, Ankara, and a voucher specimen (GUE 2604) was deposited in the Herbarium of Faculty of Pharmacy, Gazi University, Ankara, Turkey.

### 2.2. Preparation of Plant Extracts

The plant material was shade dried and powdered. Each 50 g of powdered aerial parts was submitted to successive solvent extractions separately with *n*-hexane, chloroform, ethyl acetate and methanol at room temperature for 24 h (3 × 500 ml each solvent). After filtration, the extracts were evaporated by using a rotary evaporator (Buchi, Switzerland) at 40°C to dryness *in vacuo*. Yields of each extracts were 8.3% for *n*-hexane, 17.6% for chloroform, 9.2% for ethyl acetate and 47.5% for methanol.

### 2.3. Wound Healing Activity Tests

#### 2.3.1. Animals

Male, Sprague-Dawley rats (160–180 g) and Swiss albino mice (20–25 g) were purchased from the animal breeding laboratories of Refik Saydam Central Institute of Health (Ankara, Turkey).

The animals were left for 3 days at room conditions for acclimatization. They were maintained on standard pellet diet and water *ad libitum* throughout the experiment. A minimum of six animals were used in each group, otherwise described in procedure. The study was permitted by the Institutional Animal Ethics Committee (Gazi University Ethical Council Project Number: G.U.ET-08.037) and was performed according to the international rules considering the animal experiments and biodiversity right.

#### 2.3.2. Preparation of Test Samples for Bioassay

Incision and excision wound models were used to evaluate the wound healing activity. For the *in vivo* wound models, test samples were prepared in an ointment base (vehicle) consisting of glycol stearat, 1,2 propylene glycol, liquid paraffin (3 : 6 : 1) in 1% concentration. Each test ointment (0.5 g) was applied topically on the wounded site immediately after wound was created by a surgical blade.

The vehicle group of animals was treated with the ointment base only, whereas the reference drug group of animals were treated with 0.5 g of Madecassol (Bayer, 00001199). Madecassol contains 1% extract of *Centalla asiatica*.

#### 2.3.3. Linear Incision Wound Model

All the animals were anaesthetized with 0.15 cm^3^ Ketalar and the back hair of the rats were shaved by using a shaving machine. Five-centimeter long, two linear-paravertebral incisions were made with a sterile surgical blade through the full thickness of the skin at the distance of 1.5 cm from the midline of each side of the vertebral column [21]. The wounds were closed with three surgical interrupted sutures of 1 cm apart. All the sutures used in the experiments were non-absorbable braided non-capillary and siliconized. The animals were randomly distributed into four major groups; the extracts, the reference drug the vehicle and the negative control. Six rats (160–180 g) were kept in each group. The negative control group of animals was not treated with any material, whereas the extracts, the reference drug (Madecassol) and the vehicle were applied topically once in a day throughout 9 days. All the sutures were removed on the 9th post wound day. On Day 10 all the animals were killed under anesthesia. One linear-paravertebral incised skin was measured using tensiometer (Zwick/Roell Z0.5, Germany) for its tensile strength in Newtons, the other incised skin was sent for histopathological examination [22, 23].

#### 2.3.4. Excision Wound Model

This model was employed to have information about wound contraction and wound closure time on extract applied mice compared to controls. Initially, all the animals were anaesthetized by 0.01 cm^3^ Ketalar. The back hairs of the animals were depilated by shaving. A circular wound was created on the dorsal interscapular region of each animal by excising the skin with a 5 mm biopsy punch; wounds were left open [24]. The extracts, the reference drug (Madecassol Bayer) and the vehicle were applied topically once a day on to each group (six mice) of animals, which were randomly distributed, till the wound was completely healed. The progressive changes in wound area were monitored by a camera (Fuji, S20 Pro, Japan) every other day. Later on, wound area was evaluated by using AutoCAD program. Wound contraction was calculated as percentage of the reduction in wounded area. A specimen sample of tissue was isolated from the healed skin of each group of mice for the histopathological examination [25].

### 2.4. Histopathology

The cross-sectional full-thickness skin specimens from each group were collected at the end of the experiment to evaluate for the histopathological alterations. Samples were fixed in 10% buffered formalin, processed and blocked with paraffin and then sectioned into 5 *μ*m and stained with hematoxylin & eosin (HE), Van Gieson's (VG) and toluidine blue (TB) stains [26]. Sections were analyzed and scored as mild (+), moderate (++) and severe (+++) for epidermal or dermal re-modeling. Re-epithelization or ulcus in epidermis; fibroblast proliferation, mononuclear and/or polymorphonuclear cells, neovascularization and collagen depositions in dermis were analyzed to scor the epidermal or dermal re-modeling. Van Gieson's stained sections were checked for collagen deposition and toluidine blue stained sections checked for metachromatic staining of mast cells. At the end of the examination, all the wound healing processes were combined and staged for wound healing phases as inflammation, proliferation and re-modeling in all groups.

### 2.5. Statistical Analysis of the Data

The data on percentage wound healing was statistically analyzed using one-way analysis of variance (ANOVA). The values of *P* ≤ .001 were considered statistically significant. Histopathologic data were considered to be nonparametric; therefore, no statistical tests were performed.

## 3. Results

In this study, an investigation on the *in vivo* wound healing activity of a medicinal plant, *R. sanctus* was carried out to verify the claimed traditional uses of the plant. To assess the wound healing activity of the aerial parts, extracts were prepared with different solvents; *n*-hexane, chloroform, ethyl acetate and methanol, respectively, from the aerial parts of *R. sanctus*. Incision by using tensiometer and excision wound models were employed for this activity assessment

### 3.1. Excision Wound Model

**Figure 1:**
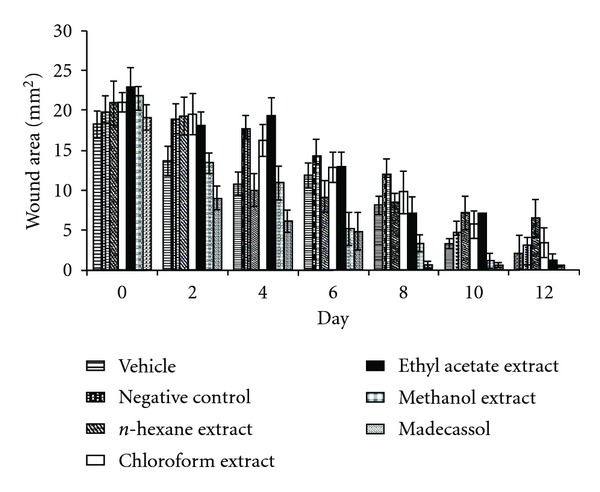
Effects of the extracts from *R. sanctus* on excision wound model.

The measurements of the progression of wound healing induced by the extracts, reference drug, negative and vehicle groups are shown in Figure 1. In this excision wound model, the methanolic extract treated groups of animals showed 56.5% contraction on the wounds at Day 6. The same extract demonstrated 80.6% contraction on the day 12, which was close to contraction value of the reference drug Madecassol (100%). However, the other extracts presented no significant results.

### 3.2. Incision Wound Model

**Figure 2:**
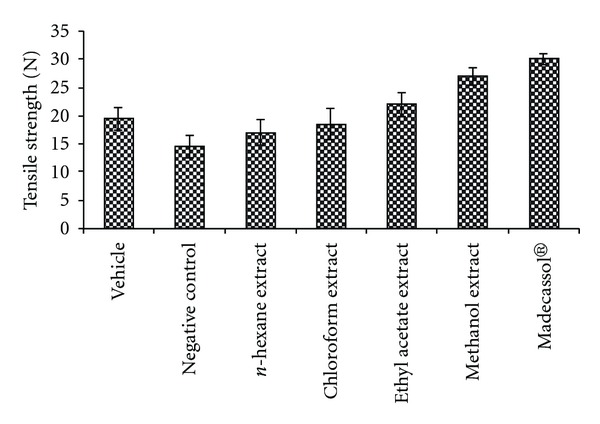
Effects of the extracts from *R. sanctus* on linear incision wound model. N: Newton.

The results of the measurements of tensile strength (in Newtons) are shown in Figure 2. Tensile strength of the animals treated with the methanolic extract demonstrated the highest value (38.9%) at day 10. Topical application of the methanolic extract on the incision wound model demonstrated a remarkable improvement in wound tensile strength compared to other groups.

### 3.3. Histopathological Examination

**Figure 3:**
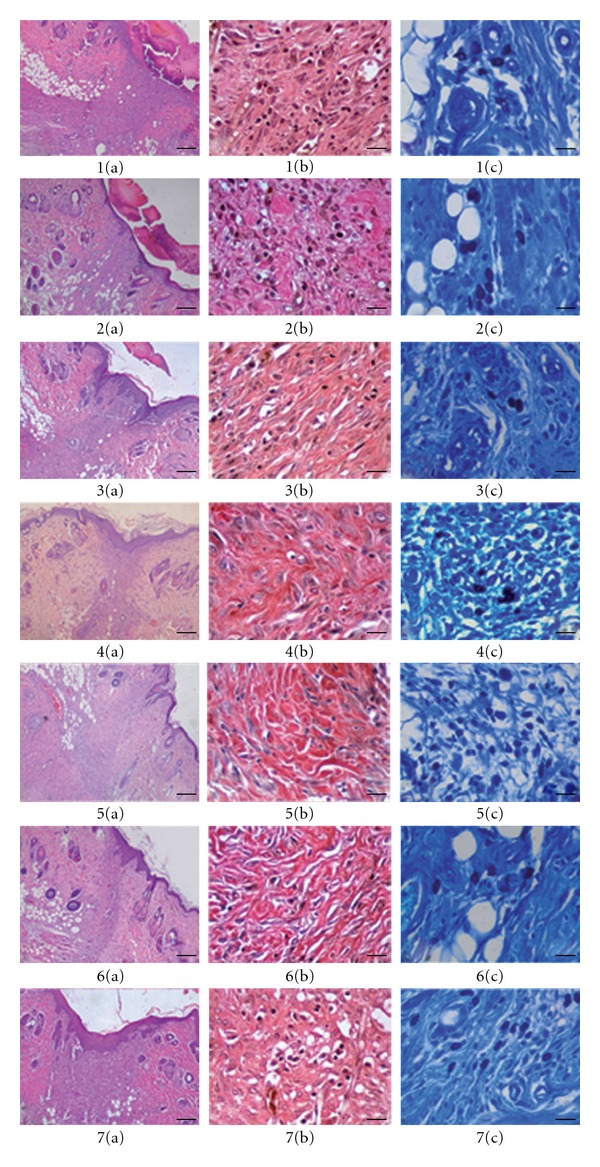
Histopathological view of wound healing and epidermal/dermal re-modeling in the vehicle, negative control, *R. sanctus* extracts and Madecassol administered animals. Skin sections show the hematoxylin & eosin (HE) stained epidermis and dermis in (a) and the dermis stained with Van Gieson's (VG) and toluidine blue (TB) in (b) and (c) respectively. The original magnification was 40× and the scale bars represent 25 *μ*m for figures in (a), and the original magnification was 400× and the scale bars represent 100 *μ*m for both (b) and (c). Data are representative of six animal per group. (i) Vehicle group, 10 days old wound tissue treated with only vehicle, (ii) negative c ontrol group (untreated), 10 days old wound tissue (iii) *n-*hexane extract group, 10 days old wound tissue treated with *n*-hexane extract, (iv) chloroform extract group, 10 days old wound tissue treated with chloroform extract, (v) ethyl acetate extract group, 10 days old wound tissue treated with ethyl acetate extract, (vi) methanol extract group, 10 days old wound tissue treated with methanol extract and (vi) Madecassol group, 10 day old wound tissue treated with Madecassol.

Following histopathological examination, evaluated, scored and staged results were combined, summarized and presented in Table 1. for demonstrating of wound healing process, representative figures (Figure 3), which stained with HE, VG and TB, were also added.

Phases in wound healing processes (inflammation, proliferation and remodeling) were observed and recorded successfully within the experimental groups (Table 1). The vehicle and the negative control groups demonstrated delayed wound healing processes compared to the other groups. In comparison with the vehicle and negative control groups, faster re-modeling were noticed in extracts treated groups. The best re-modeling, particularly, re-epithelization were detected with the methanol extract group. On the other hand, faster keratinization characterized with minor intraepithelial cornification was seen in *n*-hexane, chloroform and ethyl acetate extract groups. Weak foreign body reaction, superfluous process in wound healing, characterized with a few foreign body giant cells, which generally localized in peripheral sides of some hair follicles were detected in all groups except for the reference drug Madecassol group.

## 4. Discussion

**Figure 4:**
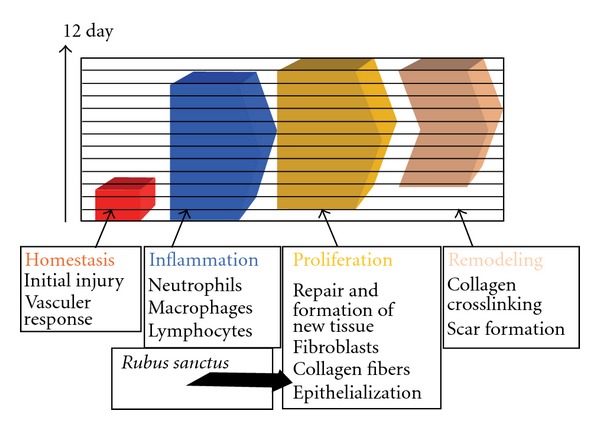
Hypothetical diagram of the wound healing mechanism of methanolic extract of *R. sanctus*

Wound healing process begins with the restoration of a damaged tissue as closely as possible to its natural state and wound contraction is the course of shrinkage in wounded area. The healing primarily depends on the repairing ability of the tissue in addition to type and degree of damage and general health status of the tissue. The granulation tissue of the wound is primarily composed of edema, fibroblast, collagen and new blood vessels. The mesenchymal cells of the wound area adjust themselves into fibroblast then begin migrating into the wound gap together with the fibrin strands. The collagen is the main constituent of extra cellular tissue, which is responsible for support and strength. Free hydroxyproline and its peptides are released with collapse of collagen. Thus, measurement of the hydroxyproline could be used as an indicator for collagen turnover. Furthermore, increase in dry tissue also indicates the presence of elevated protein content. The phytochemical analysis of aerial parts of *R. sanctus* revealed the presence of flavonoids and phenolic acid derivatives [14–16, 19]. Phenolic compounds, which include tannins and flavonoids, serve as floral pigments, structural components, feeding attractants and deterrents, of plants [27, 28]. Flavonoids have therapeutic uses due to their anti-inflammatory, anti-fungal, antioxidant and wound healing properties [29–32]. Moreover, flavonoids and their derivatives are known to decrease lipid peroxidation by improving vascularity and by preventing or slowing down the progress of cell necrosis. Hence, any drug that inhibits lipid peroxidation is supposed to increase the viability of collagen fibrils by increasing the circulation and the strength of collagen fibres, by encouraging the DNA synthesis and preventing the cell damage [33, 34]. Flavonoids [35] are also known to endorse the wound-healing process primarily due to their antimicrobial and astringent properties, which appears to be responsible for wound contraction and elevated rate of epithelization. Corresponding types of wound-healing effect were reported on medicinal plants [36, 37]. Therefore, wound-healing potential of *R. sanctus* may be attributed to the phytoconstituents present in the aerial parts, which may be either due to their individual or additive effect that speeds up the process most probably the proliferation phase of wound healing (Figure 4).

## 5. Conclusion

In conclusion, the present study demonstrated that the aerial parts of *R. sanctus* promote wound healing activity in animal as a preclinical study. The methanolic extract showed remarkable wound healing activity and it may be suggested for treating various types' wounds in animal and human beings. Further studies with purified constituents compared to the crude extracts might be needed to comprehend the complete mechanism of wound healing activity of *R. sanctus*.

## Funding

Scientific Research Project Foundation of Gazi University, Ankara, Turkey (Project code no: 02/2007-04).

## Figures and Tables

**Table 1:** Wound healing processes and healing phases of the vehicle, negative control, *R. sanctus* extracts and Madecassol administered animals^a^.

Groups	Wound healing processes									Healing phases		
	S	U	RE	FP	CD	MNC	PMN	NV	MC	I	P	R
Vehicle	+/+++	++	−/+	++	++	+	+	+/++	+	+++	++	−
Negative control	−/++	+	−/++	+/++	+/++	−/+	+	+/++	+	+++	++	−/+
Hexane extract	+	−	++	+/++	++	−/+	−	+	+	+	+/++	++
Chloroform extract	+	−	++	+/++	++	+	−	+	+	+	+/++	++
Ethyl acetate extract	+	−	++	++	++	+	−	+	+++	+	+/++	++
Methanol extract	+	−	+++	+	++	−/+	−	−/+	+	−/+	−/+	+++
Madecassol	+/++	−	++	++	++	−	−	++	+/++	+/++	++	+

S: Scab; U: Ulcus; RE: re-epithelization; FP: fibroblast proliferation; CD: collagen depositions; MNC: mononuclear cells; PMN: polymorphonuclear cells; NV: neovascularization; MC: Mast cells; I: inflammation phase; P: proliferation phase; R: re-modeling phase.

^a^HE, VG and TB stained sections were scored as mild (+), moderate (++) and severe (+++) for epidermal and/or dermal re-modeling.
